# The effect of a pharmacy-led transitional care program on medication-related problems post-discharge: A before—After prospective study

**DOI:** 10.1371/journal.pone.0213593

**Published:** 2019-03-12

**Authors:** Sara Daliri, Jacqueline G. Hugtenburg, Gerben ter Riet, Bart J. F. van den Bemt, Bianca M. Buurman, Wilma J. M. Scholte op Reimer, Marie-Christine van Buul-Gast, Fatma Karapinar-Çarkit

**Affiliations:** 1 Faculty of Health, Amsterdam University of Applied Sciences, Amsterdam, the Netherlands; 2 Department of Clinical Pharmacy, OLVG Hospital, Amsterdam, the Netherlands; 3 Department of Internal Medicine, section of Geriatric Medicine, Academic Medical Center, Amsterdam, the Netherlands; 4 Department of Clinical Pharmacology & Pharmacy, VU University Medical Center, Amsterdam, the Netherlands; 5 Community Pharmacy Westwijk, Amsterdam, the Netherlands; 6 Department of General Practice, Academic Medical Center, Amsterdam, the Netherlands; 7 Department of Pharmacy, Sint Maartenskliniek, Nijmegen, the Netherlands; 8 Department of Pharmacy, Radboud University Medical Centre, Nijmegen, the Netherlands; 9 Department of Pharmacy, University Medical Centre Maastricht, the Netherlands; 10 Department of Cardiology, Academic Medical Center, Amsterdam, the Netherlands; 11 Department of Hospital Pharmacy, BovenIJ Hospital, Amsterdam, the Netherlands; University of Mississippi, UNITED STATES

## Abstract

**Background:**

Medication-related problems are common after hospitalization, for example when changes in patients’ medication regimens are accompanied by insufficient patient education, poor information transfer between healthcare providers, and inadequate follow-up post-discharge. We investigated the effect of a pharmacy-led transitional care program on the occurrence of medication-related problems four weeks post-discharge.

**Methods:**

A prospective multi-center before-after study was conducted in six departments in total of two hospitals and 50 community pharmacies in the Netherlands. We tested a pharmacy-led program incorporating (i) usual care (medication reconciliation at hospital admission and discharge) combined with, (ii) teach-back at hospital discharge, (iii) improved transfer of medication information to primary healthcare providers and (iv) post-discharge home visit by the patient’s own community pharmacist, compared with usual care alone. The difference in medication-related problems four weeks post-discharge, measured by means of a validated telephone-interview protocol, was the primary outcome. Multiple logistic regression analysis was used, adjusting for potential confounders after multiple imputation to deal with missing data.

**Results:**

We included 234 (January-April 2016) and 222 (July-November 2016) patients in the usual care and intervention group, respectively. Complete data on the primary outcome was available for 400 patients. The proportion of patients with any medication-related problem was 65.9% (211/400) in the usual care group compared to 52.4% (189/400) in the intervention group (p = 0.01). After multiple imputation, the proportion of patients with any medication-related problem remained lower in the intervention group (unadjusted odds ratio 0.57; 95% CI 0.38–0.86, adjusted odds ratio 0.50; 95% CI 0.31–0.79).

**Conclusions:**

A pharmacy-led transitional care program reduced medication-related problems after discharge. Implementation research is needed to determine how best to embed these interventions in existing processes.

## Introduction

The incidence of medication-related problems (MRPs) ranges from 18.4% two weeks post-discharge to 37.5% four weeks post-discharge [1]. MRPs are defined as events or circumstances related to a patient’s medication [2] that can adversely affect patients’ health status [3–5]. A recent study showed that a median of 21% of hospital readmissions are due to MRPs, of which a median of 69% are regarded as preventable [6]. Examples of MRPs are the continued use of medication that had been discontinued in the hospital, side effects due to medication changes in the hospital, interactions caused by the use of home medications which were unknown during hospitalization (e.g. over-the-counter medication) or problems in implementing an altered medication regimen at home [7].

There are several causes for these post-discharge MRPs [8]. They include the often numerous changes made in medication regimens over a hospital stay [9, 10], which are not always clear to patients [11, 12]. As a consequence, patients leave the hospital insufficiently educated about the appropriate management of their altered medication regimen at home and experience difficulties in implementing this regimen [3, 5]. In addition, healthcare providers in primary care such as community pharmacists, general practitioners and home healthcare nurses are often not informed regarding medication changes and reasons for these changes and, therefore, have difficulties with monitoring a patients’ entire medication regimen [13–15]. Finally, there is no follow-up with the patient of medication-related problems that occur post-discharge.

Several pharmacy-led transitional care programs have been designed to reduce MRPs and improve the continuity of medication use by performing medication reconciliation (MR) [16–19]. MR is the process of obtaining and maintaining a complete and accurate list of the patients’ current medication use across healthcare settings [20], and has been shown to reduce MRPs during transitions in care.

Although MR is a good strategy to reduce medication errors, the tool does not intercept misunderstandings patients have about their medication, e.g. due to information overload, use of medical terms or health illiteracy. A strategy to improve patient comprehension is the “teach-back” method. This method allows healthcare providers to verify if patients and/or their family members understand discharge instructions by letting them recapitulate the information they were provided [21]. This cycle of reassessing and teaching back has been found to improve knowledge retention [22] and even lower readmission rates in heart failure patients [23]. Other post-discharge MRPs, such as side effects, interactions and problems in medication management can be addressed with post-discharge follow-up monitoring [7, 24–32]. Furthermore, the most effective interventions seem to be those that focus on collaboration between secondary and primary care incorporating a specific post-discharge strategy [1]. Until now, research on reducing MRPs has primarily focused on performing MR, either at hospital discharge or post-discharge without incorporating teach-back and connecting MR in secondary and primary care. Therefore, we designed a pharmacy-led transitional care program incorporating (i) MR at hospital admission and discharge (ii) teach-back at hospital discharge, (iii) improved transfer of medication information to primary healthcare providers and (iv) a post-discharge home visit by the patient’s own community pharmacist. This study aimed to primarily investigate the effect of this pharmacy-led transitional care program on the occurrence of MRPs four weeks post-discharge. Secondary outcomes were the number and type of interventions conducted during the home visit, patient satisfaction and patient’s knowledge of medication changes implemented during hospitalization.

## Methods

### Study design and setting

A prospective multicenter before-after study was conducted in the Netherlands, Amsterdam. In this study the departments of internal medicine, cardiology and pulmonology of a teaching hospital (OLVG) and the departments of internal medicine, cardiology, and neurology of a general hospital (BovenIJ), collaborated with 50 community pharmacies. These departments were selected as they had already implemented MR. Usual care patients were included from January through April 2016. In May and June the intervention was implemented and patients were included in the intervention group from July through November 2016. The study was approved by the local ethics committee “Adviescommissie Wetenschappelijk Onderzoek-Medisch-Ethische Commissie” (ACWO-MEC) OLVG hospital (ID WO: 15.067) and the Board of Directors BovenIJ hospital (ID WO: 5EMeh545). Written informed consent was obtained from all patients.

### Study population

Pharmacy technicians, supervised by a pharmacist-researcher, assessed patient eligibility at least 24 hours before discharge. Participants had to have spent ≥24 hours in hospital, use a minimum of three active chronic medications at discharge, defined as ≥3 prescriptions of a medicine in the previous 6 months or prescriptions for ≥90 days in a year, and had to have at least one medication change during hospitalization (excluding ‘as needed’ medication or medication prescribed for less than 5 days after discharge). These criteria were chosen because previous studies have shown that patients are prone to post-discharge MRPs if they use more than three medications and changes have been made in their medication regimen following hospitalization [3, 5]. Patients with more than one eligible admission within the duration of the study period, who were discharged to another institution (e.g. rehabilitation center or nursing home) or who could not be counseled due to physical/mental constraints, language restrictions, or those with terminal illness (as judged by their hospital physician) were excluded from the study.

### Usual care

MR was performed by (specialized) pharmacy technicians with background support of pharmacists. Pharmacy technicians have shown to perform MR accurately in the Netherlands [33]. In the Netherlands, pharmacy technicians have had a three year intermediate vocational training program that involves a combination of study in addition to practical working experience. A pharmacy technician can specialize further into pharmaceutical consultants, who have received an additional 3 year bachelor training focused on pharmacotherapy and communication [34].

For this study, the specialized pharmacy technicians were trained in executing medication reconciliation and had checklists to support them with recognizing simple medication errors, e.g. opioids without a laxative, or side effects, e.g. cough with ACE-inhibitors.

**Fig 1:**
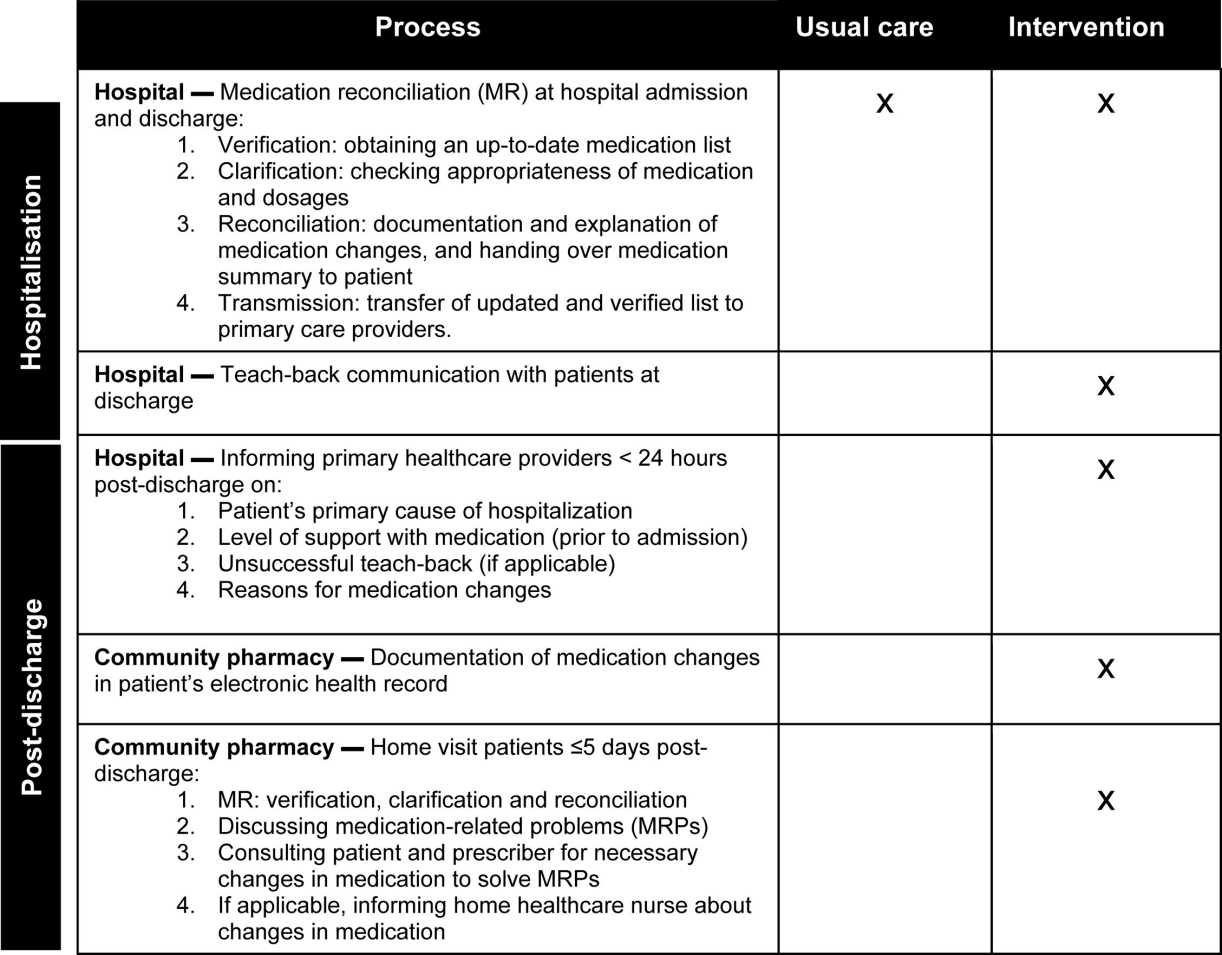
Overview of differences between usual care and intervention during hospitalization and post-discharge, and responsible party.

MR consisted of 4 steps (Fig 1) [20]. First, to gather information on actual medication use, including over-the-counter medication, community pharmacy records were collected and discussed with the patient (*verification*). In the second step, pharmacotherapy was evaluated to check whether optimization of medication was possible based on guidelines or side effects that the patient experienced (*clarification)*. In the third step, (reasons for) medication changes were documented and patients were informed of these changes (*reconciliation*). Patients were handed a personalized medication summary [34] containing information on medication (brand and generic name of medication, dose/schedule, reason for change and start or stop date), instructions about what to do in case side effects occur and clinical information (e.g. allergies, contra-indications). The pharmacy technician discussed the findings of the three steps with the hospital physician and if necessary adjustments were made in the medication regimen. Finally, the complete medication overview, including information regarding allergies, medication changes and relevant laboratory results were communicated to the community pharmacy by fax within 24 hours after discharge. This information was also added to the discharge summary to inform the general practitioner (*transmission*).

### Intervention

The interventions were added to usual care (MR during hospitalization) and were performed at discharge and post-discharge (Fig 1).

#### 1) Teach-back at discharge

Teach-back communication was added to the third reconciliation step of MR (Fig 1). Medication changes and specific care instructions (e.g. use of medication during fasting) were discussed with the patient (and/or their informal caregivers) and highlighted on their personalized medication summary. Subsequently, they were asked to ‘teach-back’ their changes in medication, either verbally or by showing it on the medication summary, to confirm patients’ understanding. Whenever these medication changes or relevant instructions were not restated correctly, information was clarified or modified and checked again, with a maximum of two reassessing and teaching back cycles. In case of unsuccessful teach-back the community pharmacy was informed, who could incorporate additional checks in patient understanding during the home visit.

#### 2) Post-discharge communication with primary healthcare providers

Within 24 hours after discharge the pharmacist-researcher informed the community pharmacist by telephone on the primary reason of hospitalization and the level of support the patient received in using medication at home prior to admission (e.g. assistance from a home healthcare nurse or informal caregiver) (Fig 1). The community pharmacist was also notified in case of unsuccessful teach-back. Finally, the patients’ medication overview, highlighting the underlying reasons for medication changes, was sent by email to the community pharmacist, home healthcare nurse and general practitioner.

#### 3) Post-discharge documentation of medication changes by community pharmacies

In the Netherlands, more than 95% of patients collect medication at only one community pharmacy [35] which registers all their medications in their individual electronic records. Previous studies have shown that even after communicating medication changes to primary healthcare providers, this information is not always documented in a patient’s electronic record [13, 36]. This inadequacy may hamper medication surveillance after hospital discharge and the continuity of pharmaceutical care. Therefore, in this study community pharmacies were instructed to also carefully document medication changes to ensure completeness of their patients’ electronic records (Fig 1).

#### 4) Home visit within five days post-discharge by community pharmacies

The main goal of this home visit, conducted by the patient’s community pharmacist, was to perform MR (Fig 1), discuss problems patients perceived with regard to their medication and focus on MRPs relevant to the patient. During this visit, medication use (indication, dosage, time of administration), knowledge of medication changes, (concerns about) side effects, doubts on effectiveness of medication, practical problems (e.g. difficulty with administration of medication due to dysphagia), medication management (e.g. multi-dose drug dispensing system) and medication adherence were discussed. Patients were also asked to allow the pharmacist to take any expired or discontinued medication for destruction. Finally, the pharmacist summarized the main points of the discussion and, if possible, directly solved all MRPs that emerged.

If changes in medication were required, the pharmacist discussed these with the patient and subsequently consulted the prescriber (either the general practitioner or hospital physician). If applicable, home healthcare nurses, who were responsible for medication administration in some patients, were informed about any medication changes. At the home visit all observations, recommendations and changes in pharmacotherapy were recorded by the pharmacist on a home visit registration form and sent to the pharmacist-researcher.

#### Training

Following the usual care period, a communication skills training was given to all pharmacy technicians about the teach-back method in the first two weeks of May, 2016. Prior to the training a communication expert observed the communication techniques of the pharmacy technicians during MR and used these observations to create the proper training. During the training session all pharmacy technicians had to practice their teach-back technique with an actor. The progress of the teach-back technique was tracked by the pharmacist-researcher who would occasionally visit a pharmacy technician during MR.

All participating community pharmacists received a one-day training from the ‘Dutch Institute for Rational Medicine Use (IVM)’ on how to appropriately conduct a home visit and deliver it in a standardized fashion, while tailoring it to patients’ needs. Information and instructions about conducting certain study elements were also given by the pharmacist-researcher, e.g. how to properly and consistently record observations in the home visit registration form.

### Data collection and outcomes

Patient characteristics (gender, age, length of stay) were extracted from the medical records of the hospital information system. The primary outcome was the difference in proportion of patients with any self-reported MRPs four weeks post-discharge, and the number of MRPs per patient, in the intervention period compared to the usual care period.

The structured telephone interview, as presented in Questionnaire A in S1 Questionnaire, was used to determine the occurrence of MRPs four weeks post-discharge and was conducted by several pharmacist-researchers. This telephone interview is based on the face- and content validated questionnaire developed by *Willeboordse et al* [37] and contains questions about medication-related symptoms, effectiveness problems or concerns, user or practical problems and remaining questions. Secondary outcomes were also assessed in the telephone interview (Questionnaire B in S1 Questionnaire):

- Recall of all medication changes implemented during hospitalization [12].
- Patient satisfaction with medication use in general and counseling during MR at discharge.
- Patient satisfaction with the post-discharge home visit (for the intervention group only).

The interventions initiated by the community pharmacist at the home visit were extracted by the pharmacist-researcher from the home visit registration form. They were classified into three categories:

1. Discrepancies: correcting unintentional differences between the documented medication in the discharge letter and actual medication use of the patient registered during the home visit.
2. Optimization of medication: any optimization of pharmacotherapy that was conducted to adhere to guidelines or reduce side effects, and
3. Patient handling interventions: improving patients’ medication use (e.g. adherence issues, problems with medication use due to dysphagia, explanation for patients’ questions).

Finally, the intervention fidelity of the study was determined to assess whether the intervention was implemented as intended [38]. This was done by measuring the adherence of healthcare professionals to the study protocol, including the number of patients who had MR during hospitalization (for both usual care and intervention group patients), teach-back at discharge (which were registered by pharmacy technicians in existing resources in the hospital), number of medication overviews transferred within 24 hours after discharge and number of home visits within five days of discharge.

### Statistical analysis

Based on results from a comparable study [26] it was estimated that a sample size of at least 200 patients per group was required to detect a decrease from 2.9 to 1.5 MRPs per patient, with a withdrawal rate of 50% (2-sided alpha test of 0.05; power of 90%;SD 2).

The primary analysis compared the proportions of patients in the two groups who reported at least one MRP four weeks post-discharge (using logistic regression analysis), and the treatment effect on the number of MRPs per patient four weeks post-discharge (using negative binomial regression analysis).

To enhance statistical stability, before conducting the latter analysis, we collapsed the sparse categories of patients with five MRPs (two patients in the intervention group and three patients in the usual care group) and six MRPs (two patients in the usual care group) into the category with four MRPs. Before the analyses of the treatment effects on the primary outcomes, we created 20 complete data sets using multiple imputation, separately for the usual care and intervention group (Stata’s multiple imputation impute chained command (by group)).

Unadjusted and adjusted analyses were performed for the primary outcomes, the latter adjusting for 14 confounders ((age (continuous; interquartile range (IQR)) 62–80), gender, ethnicity (Dutch vs non-Dutch), education (primary only, secondary, more than secondary), hospital (OLVG vs BovenIJ), type of admission (acute vs planned), length of hospital stay (continuous; IQR 4–9), number of medication at discharge (continuous; IQR 4–9), number of medication changes following hospitalization (continuous; IQR 2–5), number of medication in one of five Anatomical Therapeutic Chemical (ATC) classification groups (ordinal). The five ATC groups, concerning medication for the alimentary tract and metabolism, blood and blood forming organs, cardiovascular, nervous and respiratory system, were selected as they were the most prescribed medication in this study population and are mostly associated with the occurrence of MRPs [39, 40]. All other potential confounders were chosen as previously conducted studies have shown that these variables are associated with our primary outcome [3, 41, 42]. For example, several studies have shown that participants with a non‐western background (non-native Dutch) more often have limited literacy skills compared to native Dutch people which may be caused by difficulties in understanding information [43]. Limited literacy skills are associated with poor understanding of medications and medication changes, and this can lead to errors in medication use. Therefore, we adjusted for ethnicity in our analysis.

Furthermore, we explored if the treatment effect varied across four predetermined subgroups using treatment by subgroup interaction terms in the regression models: age (continuous), ethnicity (Dutch vs non-Dutch), number of medications at discharge (continuous), number of medication changes following hospitalization (continuous).

Treatment effects were expressed as odds ratios (OR) for logistic regression and incidence rate ratios (IRR) for negative binomial regression, and their corresponding 95% confidence intervals (CIs). Descriptive statistics were used to describe patient characteristics and secondary outcomes, and independent T tests and Chi-square tests were performed to analyze differences (p <0.05 was considered significant). All data was stored in Microsoft Excel 2010 (Microsoft, Redmond, WA) and analyzed using IBM SPSS Statistics version 21.0.0.0 (IBM Corp., Armonk, NY)) and Stata version 13.1 (Stata Corp., College Station, Texas).

## Results

### Study sample

**Fig 2:**
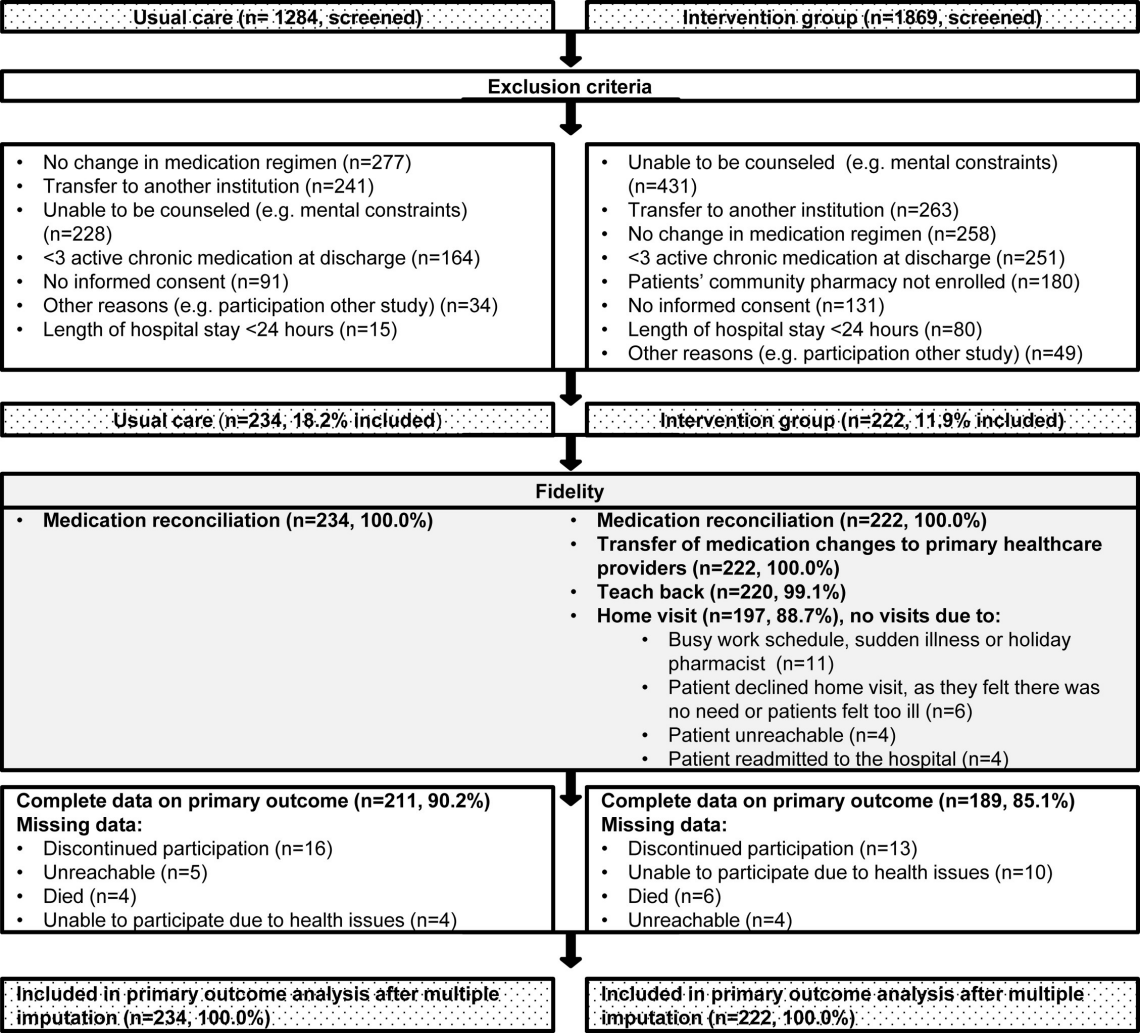
Flowchart of participants.

In total, 3153 consecutive patients were assessed for eligibility from January through November 2016. 2697 (85.5%) patients were ineligible (Fig 2). Exclusions were mainly due to the inability to counsel the patient (or informal caregiver), absence of changes in the medication regimen and transfer to other institutions following hospital discharge.

In total, 456 patients were enrolled in the study (234 in the usual care group, 222 in the intervention group). The baseline patient characteristics of both groups were essentially similar (Table 1). However, as compared to intervention patients, usual care patients lived alone more often (48.4% vs 37.4%, p = 0.02), had been hospitalized more often in the previous six months before the index admission (36.8% vs 25.9%, p = 0.01) and experienced fewer changes in their medication regimen following hospitalization (mean 3.6 vs 4.2, p = 0.01).

**Table 1:** Baseline patient characteristics.

	**Usual care (n = 234)**	**Intervention (n = 222)**	**P-value**
**Hospital of admission, n (%)**			0.14
• BovenIJ	76 (32.5)	87 (39.2)	
• OLVG, location West	158 (67.5)	135 (60.8)	
**Admission type, n (%)**			0.86
• Unplanned	213 (91.0)	201 (90.5)	
• Planned	21 (9.0)	21 (9.5)	
**Ward type, n (%)**			0.57
• Cardiology	116 (49.6)	108 (48.6)	
• Internal Medicine	67 (28.6)	59 (26.6)	
• Pulmonology	45 (19.2)	43 (19.4)	
• Neurology	6 (2.6)	12 (5.4)	
**Length of hospital stay, days, median (Interquartile range (IQR))**	6.0 (4.0–9.0)	6.0 (4.0–9.0)	0.92
**Hospitalization ≤ 6 months before index admission, n^e^ (%)**	85 (36.8)	57 (25.9)	0.01
**Gender, male, n (%)**	130 (55.6)	124 (55.9)	0.95
**Ethnicity, n (%)**			0.54
• Dutch	169 (72.2)	166 (74.8)	
• Non-Dutch	65 (27.8)	56 (25.2)	
**Age, year, mean ± SD**	70.8 ± 11.9	70.2 ± 12.8	0.58
**Living situation, n^a^ (%)**			0.02
• Alone	107 (48.4)	83 (37.4)	
• Together	114 (51.6)	139 (62.6)	
**Education^b^, n^c^ (%)**			0.57
• Primary education	94 (41.0)	97 (44.7)	
• Secondary education	97 (42.4)	82 (37.8)	
• Higher education	38 (16.6)	38 (17.5)	
**Number of medications at discharge, mean ± SD**	10.1 ± 3.9	10.2 ± 4.1	0.85
**Number of medication changes following hospitalization, mean, per patient, ± SD**	3.6 ± 2.1	4.1 ± 2.2	0.01
**Type^d^ of medication change, n (%)**			
• New	191 (81.6)	200 (90.1)	0.01
• Stop	82 (35.0)	86 (38.7)	0.41
• Dose change	98 (41.9)	74 (33.3)	0.06
• Switch	44 (18.8)	47 (21.2)	0.53

^a^ 13 missing values in usual care group.

^b^ Primary education: elementary or primary school. Secondary education: pre-vocational, senior general or pre-university. Higher education: higher professional or university.

^c^ 5 missing values in both usual care and intervention group.

^d^ Some patients had more than one type of medication change.

^e^ 3 missing values in usual care group and 1 in intervention group; information could not be provided or was missing.

### Fidelity of study protocol

Of the 234 patient included in the usual group and 222 patients in the intervention group, all received MR at hospital admission and discharge (Fig 2). For all intervention group patients the medication overview listing all medication changes and underlying reasons, was sent to primary healthcare providers. In total 220 patients received teach-back at discharge (99.1%); 38 patients (17.3%) could not successfully teach-back. In total, 197 patients (88.7%) were visited at home by their pharmacist. The remaining participants (n = 25) could not be visited due to various reasons that are specified in Fig 2. For 211 (90.2%) usual care patients and 189 (85.1%) intervention patients we had complete data on their primary outcome.

### Home visit five days post-discharge

The pharmacist spent a median of 40 minutes (IQR: 30–50 minutes) on the home visit, excluding the travel time and performed interventions after the home visit for 192 (97.5%) patients (Table 2). Pharmacists identified discrepancies for 92 patients (46.7%) in the *verification step* of MR (Fig 1). These discrepancies were mostly omission errors (38.1%), as patients often used medications that were not identified in the hospital (e.g. over-the-counter products). In the *clarification step*, pharmacists had to make changes in the medication for 43 (21.8%) patients. Mainly, dosages of medication were adjusted (e.g. increasing dose of painkillers for adequate pain-relief, reducing doses due to side effects). Finally, during the *reconciliation step*, for nearly all patients (96.4%) patient-handling interventions were conducted due to a necessity for additional education on medication (65.0%), disposal of discontinued medication (50.3%) and answering questions concerning medication (35.5%).

**Table 2:** MR interventions conducted by community pharmacist at the post-discharge home visit (n = 197).

Type of intervention	n^a^ (% of patients)
**(1) Discrepancies (start, dosage/schemes, stop, switch)**^**b**^^,^^**c**^	**92 (46.7)**
Start	74 (38.1)
Dosage regimen	24 (12.2)
Stop	4 (3.0)
Switch	1 (0.5)
**(2) Optimizations (start, dosage/schemes, stop, switch)**^**d**^	**43 (21.8)**
Dosage regimen	18 (9.1)
Stop	17 (8.6)
Start	12 (6.1)
Switch	4 (2.0)
**(3) Patient handling interventions**	**190 (96.4)**
Education about medication indication	128 (65.0)
Disposal of expired/unused medications	99 (50.3)
Answering questions concerning medication (e.g. difference between brand name and generic prescriptions, how to order new medication)	70 (35.5)
Medication compliance advice	61 (31.0)
Advice on how to reduce Medication-related problems (MRPs)	52 (26.4)
Advice on time of administration and intended duration of treatment	50 (25.4)
Advice for practical problems with medicines use (e.g. dosing aids, solutions for swallowing problems)	22 (11.2)
Advice on administration of medication (e.g. inhalation, injection)	17 (8.6)
Logistics (e.g. registering medication allergies in pharmacy information system)	15 (7.6)

^a^ Number of patients for whom at least one intervention was conducted. More than one intervention could have been conducted per patient. For example, for the subsection patient handling interventions: advice about how to increase adherence and also on administration times.

^b^ Information obtained during the home visit was considered to be the most complete and accurate.

^c^ Discrepancies were either classified as (1) Start: omission; incorrect deletion of a medication, (2) Dosage regimen: schedule of doses of a medicine, including the time between doses, the duration of treatment, the amount to be taken each time, how a medicine is to be taken, and in what dosage form. (3) Stop: commission; incorrect addition of a medication, or (4) Switch: incorrect medication.

^d^ Optimizations were either classified as (1) Dosage and scheme: dosage, administering time, medication regimen, or duration of therapy inappropriate or prescription incomplete or unclear, (2) Stop: indication no longer present, (3) Start: under treatment; medication added based on protocols and best practice standards, or (4) Switch: medication prescribed not appropriately (e.g. contraindication).

### Primary outcome four weeks post-discharge

The proportion of patients with at least one MRP four weeks post-discharge was lower in the intervention group than in the usual care group (52.4% vs 65.9%; p = 0.01) **(Table 3)**. This was mainly because of fewer symptoms caused by medication, such as gastrointestinal disorders or severe muscle pain (24.6% vs 16.4%; p = 0.04; unadjusted OR 0.60; 95% CI 0.37–0.99), and less concerns on the safety of medication use, such as fear of potential side effects (25.1% vs. 16.4%; p = 0.03; unadjusted OR 0.58; 95% CI 0.36–0.96).

**Table 3:** Patient-reported medication-related problems (MRPs) four weeks post-discharge.

	Usual care (n = 211)	Intervention (n = 189)	OR (95% CI)	P-value
**Total MRPs, n (%)**	139 (65.9)	99 (52.4)	0.57 (0.38–0.86; adjusted: 0.50 (0.31–0.79)^a^	0.01
*Number of MRPs per patient*, *mean ± SD*	1.3 ± 1.4	0.9 ± 1.1	IRR 0.70 (0.58–0.85); adjusted; 0.69 (0.55–0.86)^a^	0.00
**Type of MRP**			**OR (95% CI)**	**P-value**
**Medication-related symptoms, n (%)**	52 (24.6)	31 (16.4)	0.60 (0.37–0.99)	0.04
**Doubts, n (%)**	33 (15.6)	25 (13.2)	0.82 (0.47–1.44)	0.50
**Concerns, n (%)**	53 (25.1)	31 (16.4)	0.58 (0.36–0.96)	0.03
**Practical problems, n (%)**	44 (20.9)	35 (18.5)	0.86 (0.53–1.41)	0.56
**Difficulties, n (%)**	31 (14.7)	18 (9.5)	0.61 (0.33–1.13)	0.12
**Questions, n (%)**	47 (22.3)	26 (13.8)	0.56 (0.33–0.94)	0.03

^**a**^ After multiple imputation analysis (n = 456).

After multiple imputation, the proportion of patients with at least one MRP remained lower in the intervention group (unadjusted OR 0.57; 95% CI 0.38–0.86, adjusted OR 0.50; 95% CI 0.31–0.79). Likewise, the number of MRPs per patient was lower for patients in the intervention group than in the usual care group (mean 0.91 vs 1.32; p<0.01), also after multiple imputation (unadjusted IRR 0.70; 95% CI 0. 58–0.85, adjusted IRR 0.69; 95% CI 0.55–0.86). Finally, the subgroup analyses provided no evidence that the effect of the intervention varied among the four predefined subgroups of patients (age, ethnicity, number of medication at discharge and number of medication changes following hospitalization).

### Secondary outcome measures four weeks post-discharge

The recall rate regarding all in-hospital medication changes was higher for patients receiving the intervention compared to usual care (42.0% vs 30.2%, p = 0.01). Patients were mostly unable to recall an in-hospital discontinuation of chronic medication they used before hospital admission. Furthermore, more patients in the intervention group were satisfied with their medication use in general (82.0% vs 67.6%, p<0.01) and with MR at discharge (87.7% vs 71.1%, p<0.01) as compared to patients in the usual care group.

In total, 87.5% of patients thought the home visit was useful and that the pharmacist provided satisfactory answers to questions about their medication. Furthermore, 60.7% of patients were willing to have a second home visit by their pharmacist in case of another discharge from the hospital. Some patients (10.4%) were not sure whether a home visit was necessary and said it would depend on the number or type of changes conducted in the medication regimen during hospitalization.

## Discussion

The present pharmacy-led transitional care program was designed to reduce MRPs post-discharge. The results show that the proportion of patients with at least one MRP four weeks post-discharge was indeed 13.5% lower in the group who received a combination of hospital discharge interventions with a home follow-up visit of the community pharmacist as compared to the group who received usual care. Intervention group patients also reported less symptoms caused by their medication. These symptoms could be potential adverse drug events (ADEs) which are known to increase healthcare use [5, 44] and costs [45].

The reduction of MRPs was seen despite the high standard of usual care patients already receive during hospitalization (including MR at hospital admission and discharge), and underlines the need of additional post-discharge care. The findings show that after discharge nearly two thirds of patients in the intervention group required additional education about medication (use), such as the indication or intended duration of a treatment. This finding is consistent with previous studies [26, 46, 47]. By performing MR in the hospital, primary care providers such as community pharmacists could focus on other MRPs, such as side-effects, education on medication and a check on whether patients adhered to the medication prescribed at discharge [3, 5, 31, 48, 49]. Nevertheless, four weeks post-discharge the proportion of patients with at least one MRP remained high in the intervention group (52.4%). This rate is higher than the studies included in the review by Garcia-Caballos et al [1], in which they show the *incidence* of MRPs range from 18.4% two weeks post-discharge to 37.5% four weeks post-discharge. However, in the current study, we assessed the *prevalence* of MRPs four weeks post-discharge. Furthermore, some MRPs need more time to be resolved and not all reported MRP can be prevented or resolved. For example, patients disliked diuretics due to their mechanism of action (frequent voiding), but had an indication for diuretics (e.g. management of heart failure). However, as MRPs were self-reported by patients, overestimation of actual MRPs is possible. Still, the same MRP classification system was used for both usual care and intervention patients, and therefore we expect any aberrations of MRPs to be similar for both treatment groups. Previous studies have also identified patients’ valuable role in reporting problems due to medication [50] and have shown their ability to identify these problems [51].

The intervention, including teach-back, also improved recall rates of in-hospital medication changes. Nevertheless, still over half of intervention patients was not able to recall all medication changes post-discharge, which was also reported in other studies [11, 12]. A possible explanation may be that the recall was determined long after discharge. Moreover, the assessment took place during a telephone interview and patients were not always able to recall all changes as not all patients had their medication (overview) with them during the interview.

Overall, this pharmacy-led transitional care program is promising with high adherence to the intervention protocol (fidelity); nearly 90% of patients received the complete intervention from hospital to home. However, the economic feasibility of the intervention should also be taken into account. Not all patients are in need of this intensified type of (follow-up) care. Currently community pharmacist are not compensated for home visits that focus on medication reconciliation. However, pharmacist are compensated for home visits that incorporate medication reviews. Future research is needed to determine if pharmacy-led interventions can be embedded in existing processes in collaboration with other healthcare providers who already visit patients at home, such as home healthcare nurses who can assist in early recognition of potential MRPs.

The strengths of this study were the high fidelity to the intervention protocol, the participation of two hospitals and 50 community pharmacists, and the multiple imputation analysis to repair any impact of potentially selective drop-out on the primary outcome.

Our study had several limitations. First, patients were not randomized as contamination bias was possible at the hospital and community pharmacy level. This could dilute the real effects as the intervention intended for members of the intervention arm of a study could also be received by members of the usual care arm [26]. Second, the number of exclusions was high (86%) in both groups which might affect the generalizability of the study results. However, 55% of patients were deliberately not invited for participation because they did not fulfil inclusion criteria. Third, a before—after study design was used and therefore assessors who interviewed patients to determine MRPs could not be blinded to the exposure status of the participants. Several strategies were implemented to reduce observer bias, including the use of a standardized interview protocol, assessment by several pharmacist-researchers and a training for all assessors in how to interview patients and adequately document findings. Finally, we did not assess the effect of the intervention on clinical outcomes such as readmissions as our sample size would not be large enough to ensure adequate power. Therefore, more research is needed to study the effect of transitional care collaborations on clinical outcomes. Some promising results have already been shown in a recently published randomized trial in which a multifaceted pharmacist led intervention reduced the number of patients’ emergency department visits and hospital readmissions [52].

## Conclusion

Application of a pharmacy-led transitional care program resulted in a reduction in the proportion of patients with any self-reported MRPs, and the number of MRPs per patient, four weeks post-discharge. Follow-up care after hospitalization and close collaboration among healthcare providers across health care institutions is needed to identify, resolve and prevent MRPs and to improve the continuity of medication use. Implementation research is needed to determine how best to embed these interventions in existing processes and to determine the effect on clinical outcomes.

## Supporting information

S1 Checklist TREND statement checklist. (PDF)

S1 Questionnaire Structured telephone interview four weeks post-discharge. (DOCX)

S1 Protocol Study protocol–NON-WMO study. (PDF)

S1 Dataset Dataset. (SAV)
