# Supplementary material for: The effect of a pharmacy-led transitional care program on medication-related problems post-discharge: A before—After prospective study
Source: PLoS One. 2019 Mar 12;14(3):e0213593. doi: 10.1371/journal.pone.0213593 (PMC6413946; doi:10.1371/journal.pone.0213593)
Supplement: S1 Questionnaire — (DOCX) [file pone.0213593.s002.docx]

**S1 Questionnaire: structured telephone interview four weeks post-discharge**

**Questionnaire A: primary outcome questionnaire.**

| **Medication-related problems (MRPs)** |
| --- |
| 1. Do you experience any symptoms at the moment? *(e.g. coughing, fever, malaise, weight loss)*   - If yes*,* how many symptoms do you experience? |
| 1. Do you think the symptoms are caused by your medication?   - If yes*,* how many symptoms do you think are caused by your medication? |
| 1. Do you use medication(s) for which you have doubts that they really work for you?   *-* If yes*,* how many doubts do you have? |
| 1. Do you have concerns about your medication(s)? *(e.g. possible side effects)*   - If yes, how many concerns do you have? |
| 1. Do you have practical problems to use your medication(s)? *(e.g. difficulties with swallowing tablets)*   *-* If yes, how many practical problems do you have? |
| 1. Do you have difficulties to use your medication(s) as you physician prescribed? (*e.g. multitude of medications)*   - If yes, how many difficulties do you have? |
| 1. Do you have any additional questions about your medication(s)? *(e.g. how to order new medication)*   - If yes, how many questions do you have? |

| **Patient satisfaction with the post-discharge home visit** |
| --- |
| Please indicate whether you agree or disagree with the following statements: |
| 1. I thought the home visit was useful |
| 1. The pharmacist was able to answer all questions concerning my medication |
| 1. The pharmacist was helpful in developing a plan to suit my daily schedule |
| 1. After another discharge I would like to have a new home visit by my pharmacists |

**Questionnaire B: secondary outcomes questionnaire.**

| **Recall of all medication changes implemented during hospitalization** |
| --- |
| Were there any changes conducted in your medication regimen following hospitalisation? If yes: |
| 1. Was a new medication initiated?   - If yes, which medication(s) were newly started? |
| 1. Was a dose or frequency change initiated?   - If yes, which dose or frequency change was introduced in your medication(s)? (e.g. inhalation medication twice a day instead of once a day) |
| 1. Was a switch initiated?   - If yes, which of your medication(s) was switched? (e.g. pantoprazole to omeprazole) |
| 1. Was a medication stopped?   - If yes, which of your medication(s) was stopped? |

| **Patient satisfaction with medication use in general and counselling during medication reconciliation (MR) at discharge** |
| --- |
| 1. Were you satisfied, or neither satisfied nor dissatisfied, or dissatisfied with medication counselling at discharge? |
| 1. Are you satisfied, or neither satisfied nor dissatisfied, or dissatisfied with your medication in general? |
