# Supplementary material for: The effect of a pharmacy-led transitional care program on medication-related problems post-discharge: A before—After prospective study
Source: PLoS One. 2019 Mar 12;14(3):e0213593. doi: 10.1371/journal.pone.0213593 (PMC6413946; doi:10.1371/journal.pone.0213593)
Supplement: S1 Protocol — (PDF) [file pone.0213593.s003.pdf]

S1 Protocol: study protocol - NON-WMO study

**Title Study**	Transitional collaboration at hospital discharge to enhance continuity of care for patients
**Principal Investigator**	1. Dr. Fatma Karapinar; clinical pharmacist/epidemiologist, OLVG hospital, f.karapinar@slaz.nl
**Study sites**	1. OLVG hospital<br>2. BovenIJ hospital<br>3. Community pharmacies Amsterdam
**Short description of study:**	
Background	Different care providers are often involved in the prescription of medication, which makes pharmaceutical care complex. Care providers do not have access to the patient's entire medication file and inform each other insufficiently about changes in medication or do not know who else is involved in the patient's care process (such as other prescribers or home healthcare nurses). The patient's medication file is not managed by one care provider or by the patient himself. Finally, patients and/or informal caregivers are insufficiently informed about medication changes and do not always know which medication belongs to which disorder [1]. This fragmentation of information about medication often leads to medication errors and health damage. This particularly appears to occur during transitions (transition from one health care institution to another). In addition to medication errors, there is also a lot of inefficiency in the transition from hospital to home. Because of the communication problems at discharge, primary care providers are not always informed about changed or discontinued medication, do not know whether a high dose is consciously made or whether new medication is intended for a longer period of time (such as sleeping pills, laxatives or stomach protectors). This often requires contact with the hospital who also can not always give answers because, for example, the doctor who discharged the patient is not present and the documentation is missing. Home healthcare nurses also experience these communication problems as patients can not always tell them which medication they have to use [2]. As a result, home healthcare nurses often have to reach out to community pharmacies (which also do not have all the information and are usually closed in the evenings).<br><br>To overcome the various problems, a 'Medication transfer' guideline was developed in January 2011 in the Netherlands. This guideline had been drawn up by the Dutch Health Care Inspectorate in collaboration with various organizations. In practice, however, the guideline is applied only to a limited extent. Furthermore, the guideline is applied mostly in one setting, either in the hospital or primary care. However, to achieve an uninterrupted progression of a patient's transition from hospital to home, close cooperation between settings is necessary. In doing so, it must be ensured that the patient uses

	his or her medicines correctly.<br>In Amsterdam, the hospital pharmacies of the BovenIJ and OLVG hospitals carry out medication reconciliation, in which the medication and changes are discussed with the patient. After discharge, however, new problems may arise, such as side effects as a result of newly prescribed or changed medication [5, 6]. Kanaan et al. [7] identified adverse drug events (ADEs = harm caused by medication) in 19% of patients after discharge. Over 50% of these ADEs took place within 14 days of discharge. Coley et al. [8] found an average of 1.7 medication-related problems in 158 patients within 72 hours of discharge despite medication reconciliation at hospital discharge [8]. The authors therefore concluded that patients should receive support post-discharge. Furthermore, home healthcare nurses are often forgotten post-discharge. This research therefore focuses on actively improving the transition care between hospital and primary care and the involved care providers.
Goals of the study	Specific goals are:<br><br>- Optimizing medication reconciliation and patient education regarding medication changes in the hospital.<br><br>- Transfer of medication overviews upon discharge to the community pharmacy, general practitioner and home care.<br><br>- Improving medication use in daily life (e.g. discussing side effects or concerns about the medication) and correcting (unconscious) discrepancies with respect to discharge medication.
Method (design and outcomes)	**Design**<br><br>A before-after study will be conducted. In the before period, the hospital and the community pharmacy will provide usual care (200 patients in total). In the after period, additional interventions will take place during hospitalization, to give extra attention to changed medication among other things, and the community pharmacy will visit patients at home (200 patients in total).<br><br>**Inclusion and exclusion criteria**<br>Patients who use at least three chronic medication at discharge, are admitted ≥24 hours in hospital [3], have at least one medication change during hospitalization (excluding ‘as needed’ medication or medication prescribed for less than 5 days after discharge) and have completed the medication-reconciliation

	process in the departments of internal medicine, cardiology and pulmonology in the OLVG hospital and the departments of internal medicine, cardiology, and neurology in the BovenIJ hospital will be asked for participation. During the hospital stay patients will be asked for written informed consent and other logistical matters will be recorded (patient's phone number, preferred date for the home visit, etc.). Patients will not be included when they are transferred to another institution (e.g. nursing home), or when they have physical/mental constraints, language restrictions or terminal illness (as judged by their hospital physician).<br><br>**Before period**<br>Usual care will be provided by both hospitals in which medication reconciliation at admission and discharge will be performed by the hospital pharmacies. During the reconciliation process, pharmacy technicians verify which medication the patient uses and correct any unintended differences. The conducted medication changes during hospitalization are explained to the patient upon discharge.<br><br>The community pharmacy receives a medication overview within 24 post-discharge and guides the patient as usual (delivering medication, answering questions, etc.)<br><br>**After period**<br>The hospitals optimize the medication reconciliation process at discharge by explicitly paying attention to the explanation of medication changes (e.g. what are the reasons for these changes). During this process the teach-back method will also be applied. By using this method patients have to recapitulate the information provided regarding medication changes and given instructions. When a patient can not restate the information correctly, the information will be clarified or modified and checked again. In case of unsuccessful teach-back, the community pharmacy will be informed. Finally, patients will be asked whether they receive support from home healthcare nurses. If so, a medication overview for the home healthcare nurses will be provided by the hospital.<br><br>The community pharmacy will carefully document medication changes ≤24 hours post-discharge to ensure completeness of the patient electronic medication record and to prevent that discontinued medication is re-prescribed by other healthcare providers. Within <5 days post-discharge the community pharmacist will visit the patient at home and all observations,

	recommendations and changes in pharmacotherapy will be recorded by the pharmacist in a home visit registration form.<br><br>During this home visit pharmacists will:<br><br>- Review the patient's medication and ask them how they use the medication. Any misinterpretations or (un)intentional discrepancies will be discussed.<br><br>- Discuss the indication of medication and the embedding into daily life (adherence, side effects, for example, the patient needs help from an informal caregiver or a multi-dose drug dispensing system).<br><br>- Discuss what other medication the patient uses at home (for example, over the counter medication). Patients will also be asked to allow the pharmacist to take any expired or discontinued medication for destruction.<br><br>- Finally, the pharmacist will summarize the main points of the discussion and, if possible, directly solve all the discrepancies and observed medication-related problems.<br><br>The community pharmacist will, as care provider, ensure that the patient is not worried. When changes in medication are required, the pharmacist will discuss this with the patient and the prescriber (either the general practitioner or hospital physician). If applicable, home healthcare nurses, will also be informed about any medication changes.<br><br>**Sample size calculation**<br><br>To reduce the number of medication-related problems per patient from 2.9 to 1.5 (power 90%, alpha 0.05 and SD 2), at least 43 patients are needed per group [3]. With an expected drop of 50% (for example, because patients use less than 3 medicines or are discharged to a nursing home), at least 86 patients per group are needed. This number is rounded up to 100 patients per group per hospital (total of 400 patients). The community pharmacies will visit 200 patients in total.<br><br>**Outcomes**<br><br>- The primary outcome: all patients who participate in this study will be contacted by phone 4 weeks after discharge, using a structured telephone interview, to determine the occurrence of medication-related problems four weeks post-discharge. This telephone interview is based on the face- and content validated

questionnaire developed by Willeboordse et al. [9] and contains questions about medication-related symptoms, effectiveness problems or concerns, user or practical problems and remaining questions.

- Secondary outcomes (will also be assessed during the telephone interview): the recall of all medication changes implemented during hospitalization, patient satisfaction with medication use in general and counseling during medication reconciliation at discharge, and patient satisfaction with the post-discharge home visit.

The interventions initiated by the community pharmacist at the home visit will be extracted from the home visit registration form and will be classified into three categories:

(1) Discrepancies: correcting unintentional differences between the documented medication in the discharge letter and actual medication use of the patient registered during the home visit.

(2) Optimization of medication: any optimization of pharmacotherapy conducted to adhere to guidelines or reduce side effects, and

(3) Patient handling interventions: improving patients' medication use (e.g. adherence issues, problems with medication use due to dysphagia, explanation for patients' questions).

Finally, the intervention fidelity of the study will be determined to assess whether the intervention was implemented as intended. This will be done by measuring the adherence of healthcare professionals to the study protocol, including the number of patients who had medication reconciliation during hospitalization (for the before and after group), teach-back at discharge, number of medication overviews transferred within 24 hours after discharge and number of home visits within five days of discharge.

References

1. Karapinar-Çarkit F, et al. The effect of medication reconciliation with and without patient counseling on the number of pharmaceutical interventions among patients discharged from the hospital. Ann Pharmacother 2009;43:1001-1010.

2. Ziaeian B, et al. Medication Reconciliation Accuracy and Patient Understanding of Intended Medication Changes on Hospital Discharge. J Gen Intern Med. 2012;27:1513–1520

3. Ahmad A, et al. Identification of drug-related problems of

	elderly patients discharged from hospital. Patient Prefer Adherence. 2014;8:155-65.<br><br>4. Pasina L, et al. Medication non-adherence among elderly patients newly discharged and receiving polypharmacy. Drugs Aging. 2014;31:283-9.<br><br>5. Schoonover H, et al. Predicting potential postdischarge adverse drug events and 30-day unplanned hospital readmissions from medication regimen complexity. J Patient Saf. 2014;10:186-91.<br><br>6. Heyworth L, et al. Engaging patients in medication reconciliation via a patient portal following hospital discharge. J Am Med Inform Assoc. 2014;21:e157-62.<br><br>7. Kanaan AO, et al. Adverse drug events after hospital discharge in older adults: types, severity, and involvement of Beers Criteria Medications. J Am Geriatr Soc. 2013;61:1894-9.<br><br>8. Coley KC, et al. Pharmacist-centered hospital to home care transition initiative improves patient outcomes. Pharmacotherapy 2012;32:10(e270).<br><br>9. Willeboordse F, et al. Information on actual medication use and drug-related problems in older patients: questionnaire or interview? Int J Clin Pharm. 2016;38:380-387.
